# Possible pairing mechanism switching driven by structural symmetry breaking in BiS_2_-based layered superconductors

**DOI:** 10.1038/s41598-020-80544-2

**Published:** 2021-01-19

**Authors:** Aichi Yamashita, Hidetomo Usui, Kazuhisa Hoshi, Yosuke Goto, Kazuhiko Kuroki, Yoshikazu Mizuguchi

**Affiliations:** 1 Department of Physics, Tokyo Metropolitan University, 1-1 Minami-osawa, Hachioji, 192-0397 Japan; 2 Department of Physics and Materials Science, Shimane University, 1060, Nishikawatsucho, Matsue, 690-8504 Japan; 3 Department of Physics, Osaka University, 1-1 Machikaneyama, Toyonaka, Osaka 560-0043 Japan

**Keywords:** Phase transitions and critical phenomena, Superconducting properties and materials

## Abstract

Investigation of isotope effects on superconducting transition temperature (*T*_c_) is one of the useful methods to examine whether electron–phonon interaction is essential for pairing mechanisms. The layered BiCh_2_-based (Ch: S, Se) superconductor family is a candidate for unconventional superconductors, because unconventional isotope effects have previously been observed in La(O,F)BiSSe and Bi_4_O_4_S_3_. In this study, we investigated the isotope effects of ^32^S and ^34^S in the high-pressure phase of (Sr,La)FBiS_2_, which has a monoclinic crystal structure and a higher *T*_c_ of ~ 10 K under high pressures, and observed conventional-type isotope shifts in *T*_c_. The conventional-type isotope effects in the monoclinic phase of (Sr,La)FBiS_2_ are different from the unconventional isotope effects observed in La(O,F)BiSSe and Bi_4_O_4_S_3_, which have a tetragonal structure. The obtained results suggest that the pairing mechanisms of BiCh_2_-based superconductors could be switched by a structural-symmetry change in the superconducting layers induced by pressure effects.

## Introduction

In conventional superconductors, electron–phonon interactions are essential for the formation of Cooper pairs^1^. According to BCS (Bardeen-Cooper-Schrieffer) theory^1^, the transition temperature (*T*_c_) of a phonon-mediated superconductor is proportional to its phonon energy *ħω*, where *ħ* and *ω* are the Planck constant and the phonon frequency, respectively. Therefore, *T*_c_ of conventional superconductors is sensitive to the phonon frequency, and modifications of the isotope mass (*M*) of the constituent elements, the so-called isotope effect, have been used to investigate the importance of electron–phonon interactions in the pairing of various superconductors. The isotope exponent *α* is defined by *T*_c_ ~ *M*^−*α*^, and *α* ~ 0.5 is expected according to BCS theory^1^. For instance, *α* values close to 0.5 have been detected in (Ba,K)BiO_3_ (*α*_O_ ~ 0.5)^2^, MgB_2_ (*α*_B_ ~ 0.3)^3^, and borocarbides (*α*_B_ ~ 0.3)^4^. In addition, the hydrides (H_3_S and LaH_10_) high-*T*_c_ superconductors also showed a conventional shift in *T*_c_ with *α*_H_ = 0.3–0.5 in isotope effect investigations^5,6^. In contrast, in superconductors with unconventional mechanisms, the isotope effect is not consistent with the BCS theory, and *α* values deviated from 0.5^7,8^.

The target system of this study, layered BiCh_2_-based (Ch: S, Se) superconductors, has been extensively studied since its discovery in 2012^9–11^. Because of its layered structure composed of alternate stacking of a superconducting layer and a blocking (insulating) layer, which resembles those of high-*T*_c_ superconductors^12,13^, many studies have been performed on material development and on pairing mechanisms^11^. Although non-doped (parent) BiCh_2_-based compounds are semiconductors with a band gap, electron doping of the BiCh_2_ layers makes the system metallic, and superconductivity is induced. An example of this is F substitution in REOBiCh_2_ (RE: rare earth)^9–11^. In addition, the superconducting properties of BiCh_2_-based systems are very sensitive to the effects of external (physical) and/or chemical pressures^14–17^. When external pressures are applied, the crystal structures of REOBiCh_2_-based systems tend to distort into a monoclinic (*P*2_1_/*m*) structure, and a higher-*T*_c_ phase (*T*_c_ > 10 K) is induced^16^. Instead, by applying in-plane chemical pressure (shrinkage of the Bi-Ch conducting plane) via isovalent-element substitutions at the RE and/or Ch sites, a tetragonal (*P*4/*nmm*) phase is maintained, and bulk superconductivity is induced in the tetragonal phase. The emergence of bulk superconductivity due to chemical pressure effects can be explained by the suppression of local structural disorder, which is caused by the presence of Bi lone pair electrons^18–20^.

**Figure 1:**
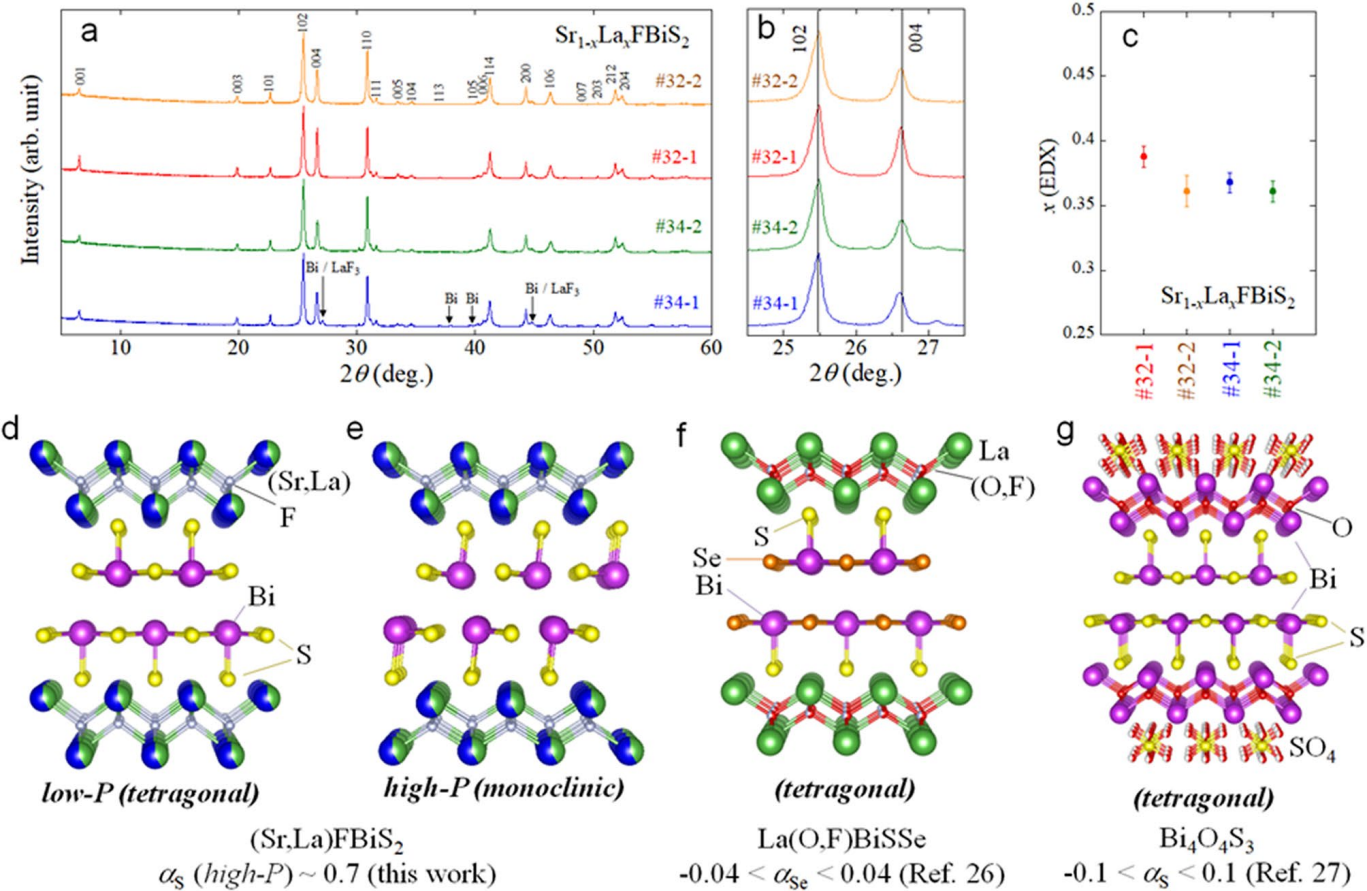
Structural and compositional data for Sr_1−*x*_La_*x*_FBiS_2_ samples with different isotope mass for sulphur. (**a**) Powder XRD patterns for #32-1, #32-2, #34-1, and #34-2. Numbers above the XRD pattern are Miller indices. Small amount of Bi and LaF_3_ impurities were detected for #34-1 as indicated by arrows. (**b**) Zoomed XRD patterns near the 102 and 004 peaks. (**c**) La concentration (*x*) analysed by EDX. (**d**–**g**) Schematic images of crystal structure of the low-*P* (tetragonal) phase and the high-*P* phase (monoclinic) of (Sr,La)FBiS_2_ and the tetragonal phase of La(O,F)BiSSe and Bi_4_O_4_S_3_. To emphasise the presence of quasi-one-dimensional network in the monoclinic phase (e), only the shorter Bi-S bonds were depicted. For comparison of the isotope effect exponent (*α*) and the crystal structure, *α*_S_ for (Sr,La)FBiS_2_, *α*_Se_ for La(O,F)BiSSe^26^, and *α*_S_ for Bi_4_O_4_S_3_^27^ (a half unit cell) are shown.

Regarding the mechanisms of superconductivity in the BiCh_2_-based family, the pairing mechanisms of the BiCh_2_-based superconductor family are still controversial^19^, owing to superconducting properties that are tunable by external and/or chemical pressure effects, which sometimes causes scattered results. Although earlier theoretical and experimental studies suggested conventional mechanisms with fully gapped s-wave pairing states^21–23^, recent theoretical calculations of *T*_c_ indicated that a *T*_c_ of an order of several K to 10 K in BiS_2_-based superconductors with a tetragonal structure cannot be explained within phonon-mediated models^24^. Furthermore, angle-resolved photoemission spectroscopy (ARPES) proposed unconventional pairing mechanisms owing to the observation of a highly anisotropic superconducting gap in NdO_0.71_F_0.29_BiS_2_^25^. In addition, a study on the Se isotope effect with ^76^Se and ^80^Se in LaO_0.6_F_0.4_BiSSe (Fig. 1f) indicated the possibility of unconventional (non-phonon) mechanisms with *α*_Se_ close to 0 (− 0.04 < *α*_Se_ < 0.04)^26^. In addition, we have recently reported on an unconventional isotope effect with ^32^S and ^34^S in Bi_4_O_4_S_3_ (− 0.1 < *α*_S_ < 0.1) (Fig. 1g)^27^. These two superconductors have a tetragonal crystal structure and show a relatively low *T*_c_ of 3.8 K for LaO_0.6_F_0.4_BiSSe and 4.7 K for Bi_4_O_4_S_3_. As mentioned above, the BiS_2_-based superconductor has a high-pressure (high-*P*) phase, which exhibits a higher *T*_c_ of over 10 K^16^. Therefore, this background encouraged us to plan an isotope effect study for a high-*P* (monoclinic) phase with a higher *T*_c_, in order to find a way to design new BiCh_2_-based superconductors with a higher *T*_c_ and to elucidate the mechanisms of superconductivity in the system.

Herein, we show experimental evidence of phonon-mediated superconductivity in a high-*P* phase of BiS_2_-based superconductors (Sr,La)FBiS_2_. We have investigated the sulphur isotope effects (^32^S and ^34^S) on *T*_c_ for a high-*P* phase of (Sr,La)FBiS_2_ with *T*_c_ ~ 10 K^28–30^. Conventional shifts in *T*_c_ between samples synthesised with ^32^S and ^34^S were observed, which suggests the importance of phonons for the pairing mechanisms in the compound. The conventional isotope effects in (Sr,La)FBiS, which has a monoclinic structure, are in contrast to the unconventional isotope effects observed in La(O,F)BiSSe and Bi_4_O_4_S_3_, which have a tetragonal structure^26,27^. Based on a combination of the discussion of previous and present isotope studies, we suggest that the structural difference between the tetragonal and monoclinic structures could be a switch of the pairing mechanisms in BiCh_2_-based superconductors.

## Results

### Characterisation of isotope samples

In general, the shift in *T*_c_ due to isotope effects is very small, even with *α* ~ 0.5 for low-*T*_c_ superconductors. Therefore, examining the isotope effects with sets of samples with comparable superconducting properties is important to reach a reliable conclusion. However, precise control of the superconducting characteristics of BiCh_2_-based compounds is the challenge of this study, because the *T*_c_ of BiCh_2_-based superconductors depends on the carrier concentration. From among the BiCh_2_-based compounds, we selected the Sr_1−*x*_La_*x*_FBiS_2_ system, because the carrier concentration in this system is essentially determined by the La concentration (*x*), and *x* can easily be analysed by compositional analysis, such as energy dispersive X-ray spectroscopy (EDX). Here, we synthesised polycrystalline samples of Sr_1−*x*_La_*x*_FBiS_2_ using ^32^S and ^34^S isotope chemicals for the investigation of sulphur isotope effects. We confirmed that the structural characteristics (particularly lattice constants) of the examined samples are comparable on the basis of powder X-ray diffraction (XRD) analyses (Fig. 1a,b). Detailed Rietveld analysis results are summarised in the Supplementary file. Although small impurity peaks of Bi and LaF_3_ were observed, the lattice constants for the examined samples were comparable as shown in Fig. 1b. The La concentration (*x*) analysed by EDX was *x* = 0.36–0.38, which is plotted in Fig. 1c. Among these samples, the carrier concentrations of samples #32-2, #34-1, and #34-2 were comparable, and that of #32-1 was slightly higher, where the sample labels indicate isotope mass (32 or 34) and batch number (1 or 2).

### Magnetisation measurements under high pressure

**Figure 2:**
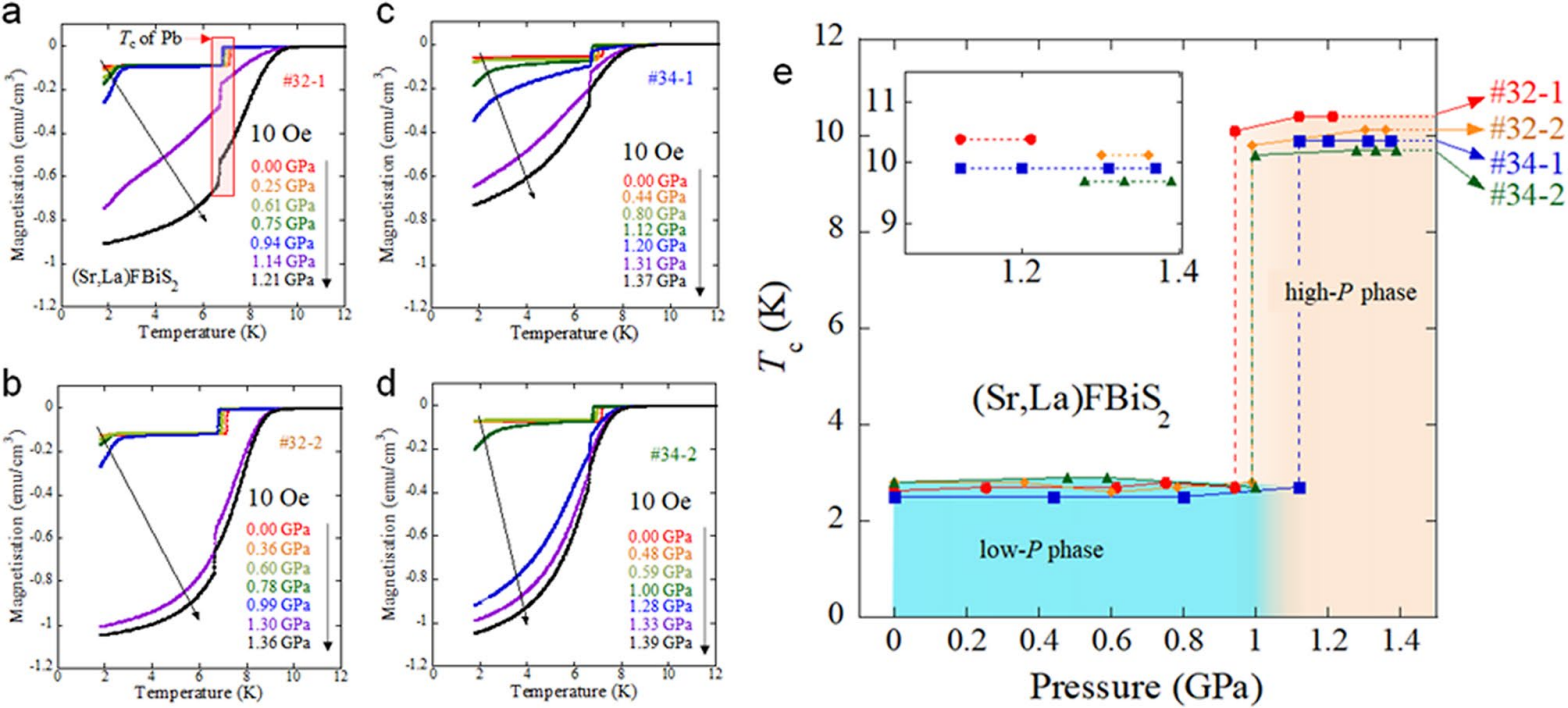
External pressure effects on the temperature dependence of magnetisation for isotope samples of Sr_1−*x*_La_*x*_FBiS_2_. (**a**–**d**) Temperature dependences of magnetisation for 32-1, #32-2, #34-1, and #34-2, respectively. Superconducting transitions at around 7 K are *T*_c_ of the Pb manometer. (**e**) Pressure dependence of *T*_c_. The inset shows the enlarged plot for the data of high-*P* phases. Note that the *T*_c_ for low-*P* phases was roughly estimated because of superconducting signals mixed with those of the high-*P* phase and the Pb manometer. See Supplementary file for the estimation of the *T*_c_ for the low-*P* phase.

**Figure 3:**
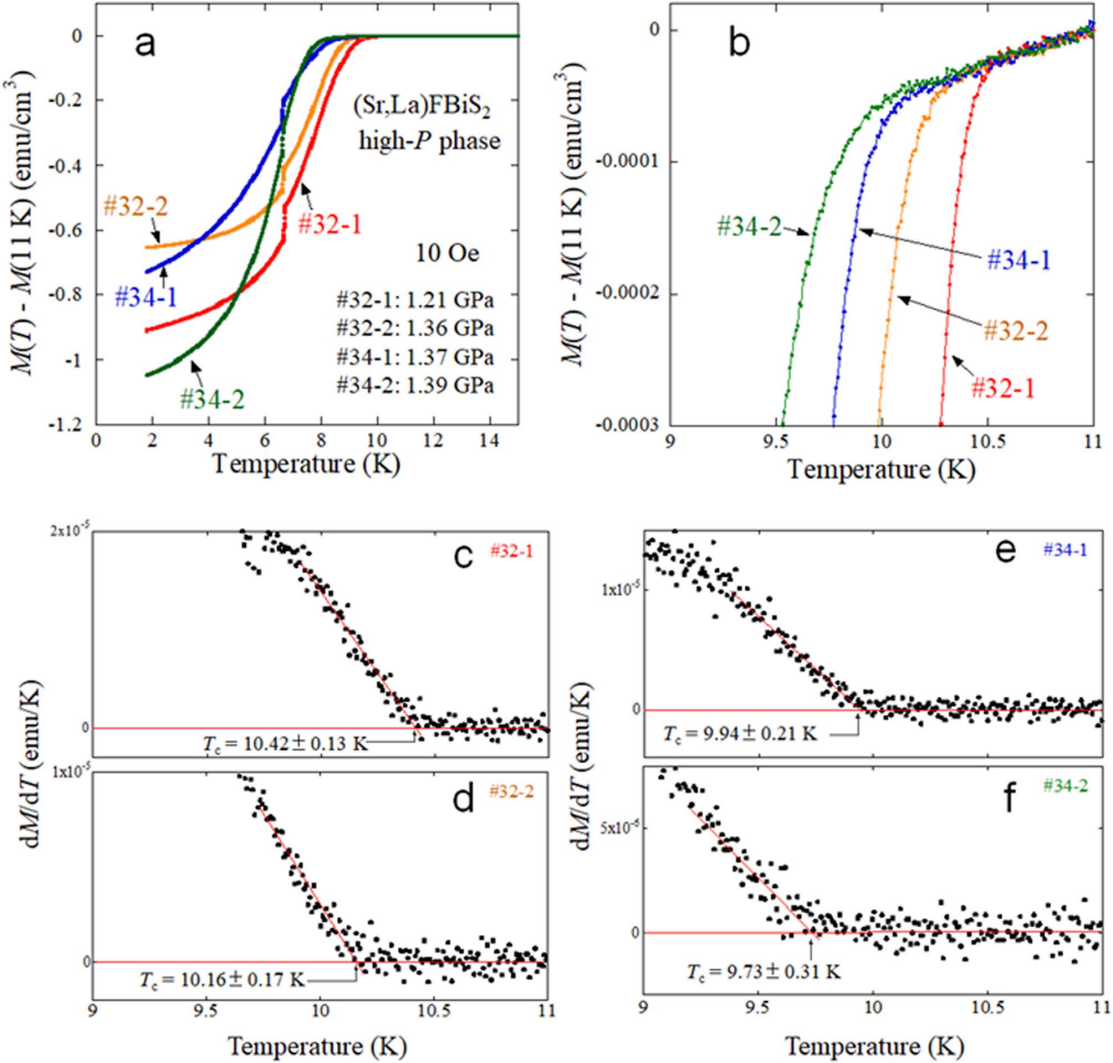
Estimation of *T*_c_^onset^ from data of the temperature dependences of magnetisation for isotope samples of Sr_1−*x*_La_*x*_FBiS_2_. (**a**) Temperature dependences of magnetisation for the high-*P* phases of 32-1, #32-2, #34-1, and #34-2. (**b**) Zoomed figure of (**a**) near the *T*_c_. (**c**–**f**) Temperature dependence of the temperature differential of magnetisation for #32-1, #32-2, #34-1, and #34-2. *T*_c_ was estimated as the temperature at which linear fitting lines of just above and just below the onset of the transition cross as indicated by the red lines in the figures.

As reported in a recent pressure study^30^, (Sr,La)FBiS_2_ shows a dramatic increase in *T*_c_ from ~ 3 K for the low-pressure (low-*P*) phase to ~ 10 K for the high-*P* phase on application of external pressure of about 1 GPa. The crystal structure of the high-*P* phase can be regarded as monoclinic, whereas that for the low-*P* phase is tetragonal, as shown in Fig. 1d,e, which is similar to the structural evolution of LaO_0.5_F_0.5_BiS_2_ under pressures^16,30^. Figure 2a–d show the temperature dependences of magnetisation measured at 10 Oe after zero-field cooling (ZFC). All samples of #32-1, #32-2, #34-1, and #34-2 show the transition from a low-*P* phase to a high-*P* phase, as plotted in Fig. 2e. Notably, in the high-*P* phase after the *T*_c_ jump, *T*_c_ does not change by an increase in applied pressure below 1.4 GPa. A similar behaviour was reported for EuFBiS_2_; the pressure dependence of *T*_c_ of EuFBiS_2_ showed a plateau under pressures above the critical pressure^31^. The appearance of the *T*_c_ plateau would be related to the structural characteristics of BiS_2_-based superconductors composed of fluoride-type blocking layers. This trend enabled us to examine the S isotope effect for the high-*P* phase of the samples. Figure 3a shows selected data of the temperature dependence of magnetisation for high-*P* phases of #32-1, #32-2, #34-1, and #34-2. Zoomed plots near the onset temperature of the superconducting transition (*T*_c_) are shown in Fig. 3b. To estimate *T*_c_, the temperature differential of magnetisation (*dM*/*dT*) was calculated and plotted as a function of temperature (Fig. 3c–f). *T*_c_ was estimated to be the temperature at which linear fitting lines for just below the transition temperature within a range of 0.5 K, as indicated by the red lines in those figures. The estimated *T*_c_ are 10.42, 10.16, 9.94, and 9.73 K for the high-*P* phases of #32-1, #32-2, #34-1, and #34-2, respectively (see Table 1 for the error). The highest *T*_c_ was observed for #32-1 with a higher La concentration (electron doping amount). For the two samples with ^34^S, *x* for #34-1 is slightly higher than *x* for #34-2, while the difference is within the error bars shown in Fig. 1c. The difference in *T*_c_, however, can be seen in Fig. 3e,f. The trend that a higher *T*_c_ is observed for a sample with higher *x* is consistent with the trend seen for #32-1 and #32-2. Although the *T*_c_ is sensitive to the La concentration, we can reach a conclusion by comparing the *T*_c_ based on the analysed La concentrations. When comparing the *T*_c_ between #32-2 and #34-1, a different trend was observed; the *T*_c_ estimated for #32-2 was higher than that of #34-1 with *x* slightly higher than *x* for #32-2. This fact implies that the isotope effect in the high-*P* phase of Sr_1−*x*_La_*x*_FBiS_2_ is conventionally present.

**Table 1:** Sample label, *x*_EDX_, and *T*_c_ for the isotope samples of Sr_1−*x*_La_*x*_FBiS_2_.

Sample label	*x*_EDX_	*T*_c_ (K)	Applied pressure (GPa)
#32-1	0.387(8)	10.42(13)	1.21
#32-2	0.361(12)	10.14(17)	1.36
#34-1	0.368(8)	9.92(21)	1.37
#34-2	0.361(8)	9.71(31)	1.39

As La concentrations for #32-2 and #34-2 are very close, estimation of their *α*_S_ may be essential, which gives *α*_S_ ~ 0.7. This value is slightly larger than the conventional *α* = 0.5 expected from BCS theory, but it suggests the importance of phonon-mediated pairing in the high-*P* phase of (Sr,La)FBiS_2_. There are uncertainties in the determination of the essential *α*_S_ for the high-*P* phase of (Sr,La)FBiS_2_ because *T*_c_ depends on the carrier concentration in this system, and the expected difference in *T*_c_ between samples with ^32^S and ^34^S is not large. However, with the results shown here and the systematic analyses of *α*_S_, we can reach the conclusion that phonons are essential for the superconductivity pairing mechanisms in the high-*P* phase of (Sr,La)FBiS_2_. This is in contrast to the unconventional isotope effects observed in La(O,F)BiSSe^26^ and Bi_4_O_4_S_3_^27^. We discuss the possible differences in the structural and electronic characteristics of (Sr,La)FBiS_2_ (*α*_S_ ~ 0.7) and La(O,F)BiSSe (− 0.04 < *α*_Se_ < 0.04)^26^ in the following section.

## Discussion

As summarised in Fig. 1, isotope effect suggesting the importance of phonon was observed for the high-*P* phase of (Sr,La)FBiS_2_, whereas unconventional isotope effects were observed in La(O,F)BiSSe and Bi_4_O_4_S_3_^26,27^. Although there are some possible factors, which could affect isotope effect, other than pairing states^32^, we consider that the observed difference in isotope effect is essentially caused by the different pairing states between those systems. The reason for proposing the scenario is the recent observation of nematic superconductivity in La(O,F)BiSSe^33,34^; nematic superconductivity has been observed in unconventional superconductors like Fe-based and Bi_2_Se_3_-based superconductors^35,36^. Since nematic superconductivity emerges in both LaO_0.9_F_0.1_BiSSe (tetragonal) and LaO_0.5_F_0.5_BiSSe (tetragonal) with different carrier concentrations but with comparable structures of the BiSSe conducting layer, unconventional pairing states would commonly present in tetragonal BiCh_2_-based superconductors with a tetragonal symmetry without structural distortion or local disorder. In contrast, nematic superconductivity was not observed in Nd(O,F)BiS_2_^37^, which is also tetragonal but has larger local structural disorder than La(O,F)BiSSe^11,17,19^. These facts suggest the importance of structural symmetry in the conducting layers and would support our scenario suggested in this article. Here, we discuss the possible differences in electronic states and pairing states between tetragonal and monoclinic phases.

The high-*P* phase of (Sr,La)FBiS_2_ has a monoclinic structure and a distorted in-plane structure in the BiS_2_ layers^30^. In contrast, La(O,F)BiSSe and Bi_4_O_4_S_3_ have tetragonal structures, in which the square Bi-Ch network forms a superconducting plane. Although the low-*P* phase of (Sr,La)FBiS_2_ is tetragonal, same as for La(O,F)BiSSe and Bi_4_O_4_S_3_, bulk superconductivity is not observed at ambient pressure because of insufficient in-plane chemical pressure^17–19,30^. In the low pressure range, bulk superconductivity is induced, but the determination of *T*_c_ is difficult because there are two possible superconducting transitions of the manometer (Pb) and the high-*P* phase. Based on the isotope effects in the high-*P* phase of (Sr,La)FBiS_2_, La(O,F)BiSSe, and Bi_4_O_4_S_3_, we suggest that structural symmetry breaking in the superconducting BiCh_2_ layer is an essential factor in the switching of the isotope effect from unconventional to conventional.

We calculated the band structures of (Sr,La)FBiS_2_ and La(O,F)BiSSe (see Supplementary file). Note that the calculated results for (Sr,La)FBiS_2_ are based on the tetragonal structure of the low-*P* phase, because structural parameters for the high-*P* phase have not been experimentally obtained for (Sr,La)FBiS_2_, and the structural relaxation was not successful for the high-*P* phase in this work. One can determine that the shape of the Fermi surface is similar between (Sr,La)FBiS_2_ and La(O,F)BiSSe, because the expected carrier doping amount is comparable. Therefore, we consider that the different isotope effects were due to the modifications of electronic and/or phonon characteristics induced by structural symmetry breaking in the monoclinic phase. According to previous theoretical calculations for the tetragonal and monoclinic phases of La(O,F)BiS_2_, band splitting results from a structural transition from tetragonal (low-*P* phase) to monoclinic (high-*P* phase)^38^. In addition, the impact of interlayer coupling between two BiS_2_ layers, caused by the structural symmetry breaking, on the electronic states was suggested as a possibility. The switching of isotope effects between the tetragonal and monoclinic phases may be linked to the formation of the Bi–Bi bonding in the high-*P* phase in the present system. Let us remind that the theoretical study on the calculation of *T*_c_ for LaO_0.5_F_0.5_BiS_2_ by Morice et al.^24^ was performed on a tetragonal unit cell. Their conclusion is consistent with the unconventional isotope effects observed in tetragonal La(O,F)BiSSe and Bi_4_O_4_S_3_. If the same calculation of *T*_c_ could be performed on a monoclinic unit cell, a *T*_c_ of 10 K may be reproduced. For that, high-resolution structural analyses of the high-*P* phase of (Sr,La)FBiS_2_ are needed.

In conclusion, we synthesised (Sr,La)FBiS_2_ polycrystalline samples with ^32^S and ^34^S isotope chemicals. With magnetisation measurements under high pressure, we investigated the sulphur isotope effects on *T*_c_ for a high-*P* phase of (Sr,La)FBiS_2_. As a conventional shift in *T*_c_ was observed, we suggested the importance of phonons for the pairing mechanisms for the high-*P* phase. Based on comparisons with isotope effects in La(O,F)BiSSe and Bi_4_O_4_S_3_, in which unconventional isotope effects have been observed, we suggest that structural symmetry breaking from tetragonal to monoclinic is a key factor for the switch of the isotope effects in the BiCh_2_-based superconductor family.

## Methods

Polycrystalline samples of (Sr,La)FBiS_2_ were prepared by a solid-state reaction method in an evacuated quartz tube. Powders of La (99.9%), SrF_2_ (99%), and Bi (99.999%) were mixed with powders of ^32^S (ISOFLEX: 99.99%) or ^34^S (ISOFLEX: 99.26%) with a nominal composition of Sr_0.5_La_0.5_FBiS_2_ in an Ar-filled glove box. The mixed powder was pelletised, and sintered in an evacuated quartz tube at 700 °C for 20 h, followed by furnace cooling to room temperature. The obtained compounds were thoroughly mixed, ground, and sintered under the same conditions as the first sintering. Except for the starting materials, the synthesis method was the same as our recent study on (Sr,La)FBiS_2_^30^.

The phase purity and crystal structure of the (Sr,La)FBiS_2_ samples were examined by laboratory X-ray diffraction (XRD) by the *θ*-2*θ* method with Cu-Kα1 radiation on a SmartLab (RIGAKU) diffractometer. The schematic images of crystal structures were drawn by VESTA^39^ using structural data refined by Rietveld refinement using RIETAN-FP^40^. Through the XRD analyses, Bi and LaF_3_ impurity phases were detected. The actual compositions of the examined samples were analysed using energy dispersive X-ray spectroscopy (EDX) on a TM-3030 instrument (Hitachi). The average value of *x*_EDX_ was calculated using the data obtained for five points on the sample surface. Standard deviation was estimated and shown in Table 1. Through the XRD analyses, small spots with La-rich compositions were found. The impurity phase will be LaF_3_, since a LaF_3_ phase was found in XRD.

The temperature dependence of the magnetisation at ambient pressure and under high pressures was measured using a superconducting quantum interference device (SQUID) on MPMS-3 (Quantum Design) after zero-field cooling (ZFC). Hydrostatic pressures were generated by the MPMS high-pressure capsule cell made of nonmagnetic Cu-Be, as described in our recent high pressure study on (Sr,RE)FBiS_2_^30^. The sample was immersed in a pressure transmitting medium (Daphene 7373) and covered with a Teflon cell. The pressure at low temperature was calibrated based on the superconducting transition temperature of the Pb manometer. The magnetisation data shown in this paper contains background magnetisation. For sample #32-1, the maximum pressure was lower than that for other samples, which is due to the setup of high-pressure cell with a shorter piston stroke.

## Supplementary information

Supplementary Information.

## Data Availability

All relevant data are available from the corresponding authors upon reasonable request.
