# Supplementary material for: Possible pairing mechanism switching driven by structural symmetry breaking in BiS2-based layered superconductors
Source: Sci Rep. 2021 Jan 19;11:230. doi: 10.1038/s41598-020-80544-2 (PMC7815720; doi:10.1038/s41598-020-80544-2)
Supplement: Supplementary file 1 — Supplementary Information. [file 41598_2020_80544_MOESM1_ESM.pdf]

Supplementary file

Possible pairing mechanism switching driven by structural symmetry breaking in BiS_2-based layered superconductors

Aichi Yamashita[1], Hidetomo Usui[2], Kazuhisa Hoshi[1], Yosuke Goto[1], Kazuhiko Kuroki[3], Yoshikazu Mizuguchi[1]*

[1]*Department of Physics, Tokyo Metropolitan University, 1-1, Minami-osawa, Hachioji 192-0397, Japan.*

[2]*Department of Physics and Materials Science, Shimane University, 1060, Nishikawatsucho, Matsue 690-8504, Japan.*

[3]*Department of Physics, Osaka University, 1-1 Machikaneyama, Toyonaka, Osaka 560-0043, Japan.*

Corresponding author: Yoshikazu Mizuguchi (mizugu@tmu.ac.jp)

Table S1. Rietveld refinement results for the examined $Sr_{1-x}La_xFBiS_2$ samples with ^{32}S and ^{34}S isotopes. In the refinements, a single-phase analysis mode was used, and the La concentration x was fixed as the value determined by EDX.

Label	#32-1	#32-2	#34-1	#34-2
x (EDX)	0.387(8)	0.361(12)	0.368(8)	0.361(8)
Space group	Tetragonal *P*4/*nmm* (#194)			
a (Å)	4.084 (3)	4.084(4)	4.084(3)	4.083(4)
c (Å)	13.352(11)	13.353(13)	13.366(11)	13.345(12)
z (Sr,La)	0.1108(2)	0.1109(2)	0.1141(3)	0.1133(3)
z (Bi)	0.62368(14)	0.6226(2)	0.6256(2)	0.6245(2)
z (S1)	0.3703(10)	0.3780(9)	0.3638(12)	0.3692(12)
z (S2)	0.8165(6)	0.8112(6)	0.8192(7)	0.8156(7)
R_{wp} (%)	14.1	13.0	15.9	13.1

[Atomic coordinates]

(Sr,La): (0, 0.5, z)

F: (0, 0, 0)

Bi: (0, 0.5, z)

S1 (in-plane site): (0, 0.5, z)

S2 (out-of-plane site): (0, 0.5, z)

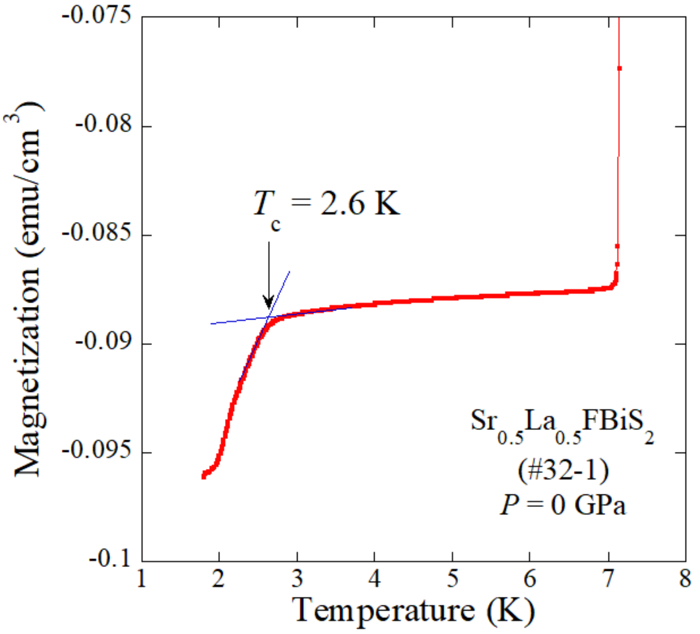

Fig. S1. Estimation of T_c for the low-*P* phase from the temperature dependence of magnetization for #32-1 at ambient pressure.

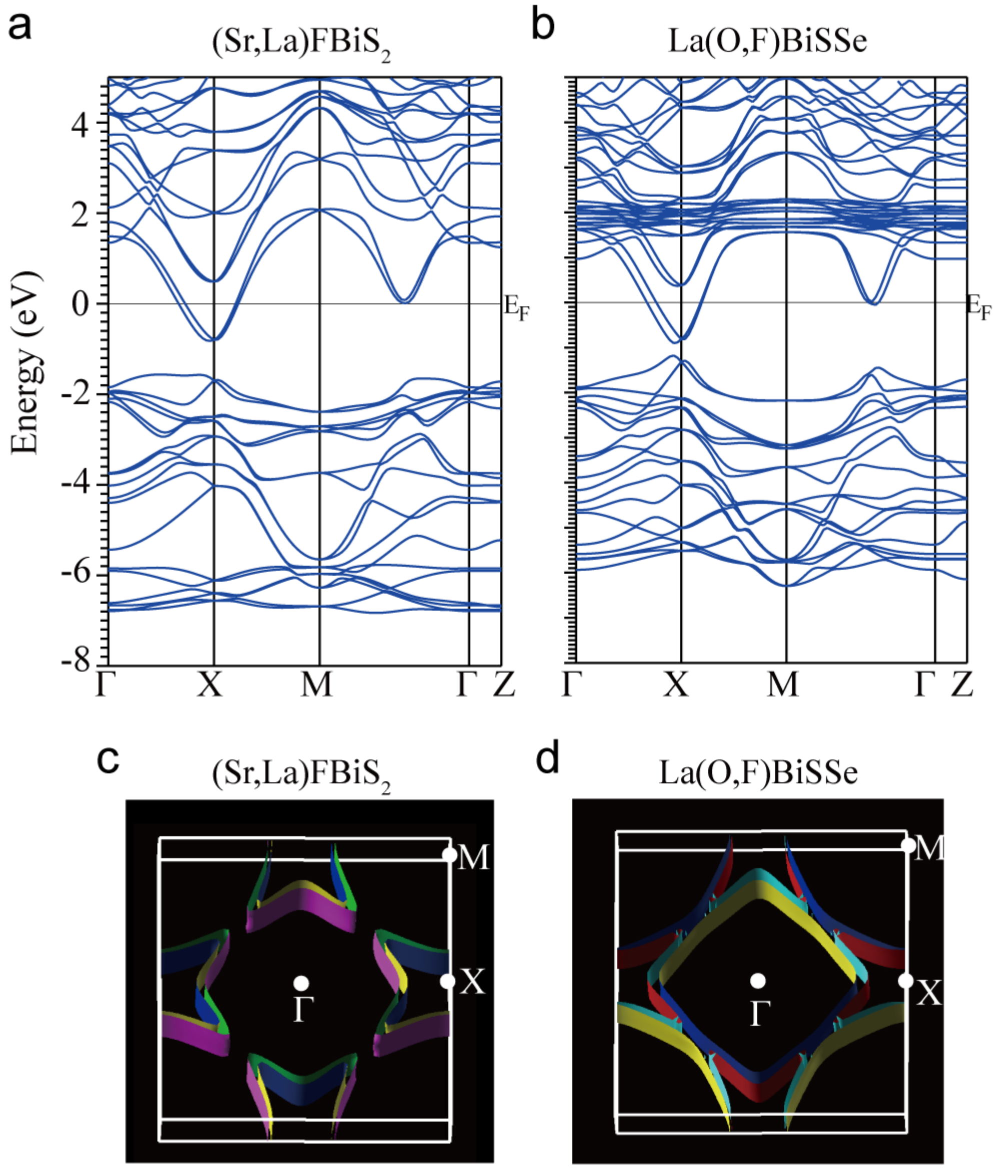

Fig. S2. Calculated band structure for $Sr_{0.6}La_{0.4}FBiS_2$ (tetragonal) and $LaO_{0.6}F_{0.4}BiSSe$ (tetragonal). (a, c) Calculated electronic band structure and Fermi surface for $Sr_{0.6}La_{0.4}FBiS_2$. The band calculations were performed from a (Ba,La)$FBiS_2$ model with a lattice structure of $Sr_{1-x}La_xFBiS_2$ with x = 0.4. (b, d) Calculated electronic band structure and Fermi surface for $LaO_{1-x}F_xBiSSe$ with x = 0.4.

Methods for band calculations

First-principles band calculations for $Sr_{1-x}La_xBiS_2$ and $LaO_{1-x}F_xBiSSe$ were performed using the WIEN2k package[1,2]. We used a virtual crystal approximation to simulate partial substitution. Because of technical reasons concerning the virtual crystal approximation, we used Ba instead of Sr for (Sr,La)$FBiS_2$. In the WIEN2k package, the virtual crystal approximation can be performed for elements adjacent to each other in the periodic table. We calculated the electronic band structure of (Sr,La)$FBiS_2$ using the VASP package[3,4], assuming virtual crystal approximation. We have confirmed that the electronic band structure is not strongly affected by the replacement of Sr by Ba in the calculations. The electronic band structures of $Ba_{0.6}La_{0.4}FBiS_2$ and $LaO_{0.6}F_{0.4}BiSSe$ were obtained by adopting the experimental lattice constants of $Sr_{1-x}La_xFBiS_2$ (#32-2: parameters shown in Table S1) and $LaO_{0.6}F_{0.4}BiSSe$[5], respectively. We used $RK_{max} = 7$ and a 18×18×5 k-mesh for self-consistent calculations, and adopted the Perdew-Burke-Ernzerhof exchange-correlation functional[6] including the spin-orbit coupling.

1. Blaha, P. *et al.* WIEN2k, An Augmented Plane Wave + Local Orbitals Program for Calculating Crystal Properties (Karlheinz Schwarz, Techn. Universität Wien, Austria), 2018. ISBN 3-9501031-1-2.
2. Blaha, P. *et al.* WIEN2k: An APW+lo program for calculating the properties of solids. *J. Chem. Phys.* 152, 074101 (2020).
3. Kresse, G. & Furthmüller, J. Efficient iterative schemes for ab initio total-energy calculations using a plane-wave basis set. *Phys. Rev. B* 54, 11169 (1996).
4. Kresse, G. & Joubert, D. From ultrasoft pseudopotentials to the projector augmented-wave method. *Phys. Rev. B* 59, 1758 (1999).
5. Hoshi, K., Goto, Y., & Mizuguchi, Y. Selenium isotope effect in the layered bismuth chalcogenide superconductor $LaO_{0.6}F_{0.4}Bi(S,Se)_2$. *Phys. Rev. B* 97, 094509(1-5) (2018).
6. Perdew. J. P., K. Burke & Ernzerhof, M. Generalized Gradient Approximation Made Simple. *Phys. Rev. Lett.* 77, 3865–3868 (1996).
